# Participant experiences of Eye Movement Desensitisation and Reprocessing vs. Cognitive Behavioural Therapy for grief: similarities and differences

**DOI:** 10.1080/20008198.2017.1375838

**Published:** 2017-10-09

**Authors:** Prudence Cotter, Larissa Meysner, Christopher William Lee

**Affiliations:** ^a^ Department of Psychology and Exercise Science, Murdoch University, Perth, Australia; ^b^ Faculty of Health and Medical Sciences, University of Western Australia, Perth, Australia

**Keywords:** Grief, Eye Movement Desensitisation and Reprocessing, Cognitive Behavioural Therapy, qualitative methods, dolor, Desensibilización y reprocesamiento mediante movimientos oculares, terapia cognitivo conductual, métodos cualitativos, 哀痛, 眼动脱敏与再加工, 认知行为疗法, 质性方法

## Abstract

**Background and Objective**: Previous research has used quantitative methods to assess the impact of grief therapy. However, rarely have participants been asked about how they have been affected by treatment using qualitative methods. This study sought to explore participants’ experiences of two therapeutic approaches to grief: Cognitive Behavioural Therapy (CBT) and Eye Movement Desensitisation and Reprocessing (EMDR).

**Method**: Nineteen participants were randomly allocated to receive seven weekly therapy sessions of either CBT or EMDR. Approximately two weeks after completing therapy, a semi-structured interview was conducted with each participant. Interviews were transcribed and a thematic analysis was performed.

**Results**: Participant reports common to both therapies included developments in insight, a positive shift in emotions, increased activity, improved self-confidence and a healthier mental relationship to the deceased. Participants also responded by describing experiences that were unique to each therapy. Those who completed CBT described the acquisition of emotion regulation tools and shifting from being in an ongoing state of grief to feeling that they were at a new stage in their lives. Participants who completed EMDR reported that distressing memories were less clear and felt more distant from such memories following treatment.

**Conclusions**: Although both therapies resulted in some similar changes for participants, there were unique experiences associated with each therapy. These findings are discussed in terms of implications for the underlying key processes of each therapy and the processes of recovery in grief.

## Abstract (es)

**Planteamiento y objetivos:** Las investigaciones previas han utilizado métodos cuantitativos para evaluar el impacto de la terapia de duelo. Sin embargo, rara vez se han empleado métodos cualitativos para preguntar a los participantes cómo se han visto afectados por el tratamiento. Este estudio trató de explorar las experiencias de los participantes con dos enfoques terapéuticos sobre duelo: la terapia cognitivo conductual (TCC) y la desensibilización y reprocesamiento mediante movimientos oculares (EMDR, siglas en inglés de *Eye Movement Desensitisation and Reprocessing*)

**Métodos:** Diecinueve participantes fueron asignados al azar para recibir siete sesiones de terapia semanal de TCC o EMDR. Aproximadamente dos semanas después de completar el tratamiento, se llevó a cabo una entrevista semiestructurada con cada participante. Se transcribieron las entrevistas y se realizó un análisis temático.

**Resultados:** Los informes de los participantes que fueron comunes a ambas terapias incluyeron avances en las auto-percepciones, un cambio positivo en las emociones, un aumento de la actividad, una mayor confianza en sí mismos y una relación mental más sana con el fallecido. Los participantes también respondieron describiendo experiencias que eran únicas para cada terapia. Aquellos que completaron la TCC describieron haber adquirido herramientas de regulación emocional y haber pasado de estar en un estado de dolor continuo a sentir que estaban en una nueva etapa en su vida. Los participantes que completaron EMDR indicaron que los recuerdos angustiosos eran menos claros y que se sentían más distantes de tales recuerdos después del tratamiento.

**Conclusiones:** Aunque ambas terapias dieron como resultado algunos cambios similares para los participantes, hubo experiencias únicas asociadas con cada terapia. Estos hallazgos se discuten en términos de implicaciones para los procesos claves subyacentes de cada terapia y los procesos de recuperación en el proceso de duelo.

## Abstract (zh)

**背景和目标：**过去研究使用量化研究方法评估哀痛疗法的影响。但质性方法很少被用于询问参与者的体验。本研究旨在探索参与者在两个治疗哀痛的方法中的体验：认知行为俩佛奥（CBT）和眼动脱敏与再加工（EMDR）。

**方法：**19名参与者随机分配到CBT或EMDR的7周疗程。完成治疗的差不多两周后，对每个参与者进行半结构访谈。访谈内容被转录后进行主题分析。

**结果：**两种疗法的参与者都普遍报告了悟性提高、情绪的正向改变、活动性提高、自信心增加和对逝者更健康的心理联结。参与者也报告了两种疗法的独特性。完成CBT的参与者描述获得了情绪调节工具，并能从哀痛中转移到体验到人生新阶段。完成EMDR的参与者报告痛苦记忆变得较不清楚，并感到和这些记忆隔得较远。

**结论：**尽管两种疗法对参与者的一些改变相似，也带来了和疗法相关的独特体验。我们讨论这些发现以发现每种疗法背后的关键过程和哀痛恢复的过程。

## HIGHLIGHTS

- Participants in both the EMDR and CBT groups reported positive changes in their experience of grief and quality of life following treatment.
- Some of the changes described were unique to each treatment condition, and these aligned with the underlying theories of EMDR and CBT respectively.
- CBT participants reported acquiring tools and skills to regulate emotions.
- EMDR participants reported an increased distancing from their upsetting memories.

Grief following the death of a loved one is an almost universal experience; however, for some the pain and anguish that follows such a loss does not subside and continues to impact functioning long after the death. There have been attempts to distinguish between normal grief and a collection of symptoms that would warrant a diagnostic label. The most current labels are Persistent Complex Bereavement Disorder (PCBD) as defined in the fifth edition of the Diagnostic and Statistical Manual of Mental Disorders (American Psychiatric Association, 2013) and Prolonged Grief Disorder (PGD) which has been proposed for inclusion in the 11th revision of the International Classification of Diseases (ICD-11; Maciejewski, Maercker, Boelen, & Prigerson, 2016). These diagnostic classifications are met when the grief reaction is prolonged and accompanied by symptoms such as yearning for the deceased, preoccupation with the deceased or their death, and difficulty accepting the loss. Maciejewski et al. (2016) suggested that there are no substantive differences between PGD and PCBD and that each is associated with a prevalence rate of around 10%. Issues surrounding the controversy over a proposed diagnostic criteria for grief (either PGD or PCBD) are the potential for pathologizing what may be considered normal reactions to loss. Another issue is that a focus on a diagnosis such as PCBD may not capture all the psychological morbidity following the death of a loved one (Boelen, 2016).

The findings from meta-analyses of grief interventions imply that individuals who meet a diagnostic criteria of grief symptoms will benefit substantially from therapy, compared to bereaved individuals experiencing ‘normal’ grief (Wittouck, Van Autreve, De Jaegere, Portzky, & van Heeringen, 2011). Whereas in other reviews (Allumbaugh & Hoyt, 1999; Schut, Stroebe, van den Bout, & Terheggen, 2001), consistently larger effect sizes and positive outcomes have been reported where participants were self-referred or referred by a general practitioner, when compared to preventative or outreach approaches. These studies suggest that a self-perception of struggling with grief and active treatment seeking results in a benefit from therapy.

One treatment that has been shown to be effective in reducing symptoms associated with grief is cognitive behavioural therapy (CBT). It involves teaching participants skills to initially identify and then modify unhelpful thoughts and behaviours (Boelen, van den Hout, & van den Bout, 2006). CBT for grief is underpinned by the theory that problematic grief symptoms are maintained by negative cognitions and avoidant behaviours. CBT has been found to lead to larger and more rapid grief symptom reduction compared to interpersonal therapy (Shear, Frank, Houck, & Reynolds, 2005).

Another approach to treating grief is Eye Movement Desensitisation and Reprocessing (EMDR), which was designed to deal with traumatic memories (Shapiro, 1995). In this treatment, the therapist facilitates the client to access the critical components of their distressing memories while simultaneously engaging in another task, such as eye movements. This therapeutic process has been found to result in a reduction in Posttraumatic Stress Disorder (PTSD) symptoms, such as the frequency of intrusive symptoms (Chen et al., 2014), and a reduction in the vividness and emotional intensity of distressing memories (Lee & Cuijpers, 2013). While EMDR has been systematically studied as a therapy for PTSD, there is very little research on its effects on grief. In a non-randomized controlled study, Sprang (2001) found that an EMDR group required significantly fewer sessions compared to the control condition of ‘guided mourning’ to achieve the same symptom reduction. In another study, participants who reported distressing memories following the death of a loved one (Hornsveld et al., 2010) had greater decline in emotionality of a grief-related memory following a recall plus eye movements condition compared to recall-only or recall with music condition.

In a randomized controlled trial, CBT was compared to EMDR for individuals who had reported they needed help with grief (Meysner, Cotter, & Lee, 2016). Following a wait-list control, participants who had lost a loved one received 10 hours of either CBT or EMDR. Both treatment groups improved on measures of trauma and grief symptomology with no significant differences between the treatments on the primary outcome measures.

The above study found no differences in quantitative measures following treatment but that does not mean that participants experienced each therapy in the same way or that the same processes were involved in each therapy. Furthermore while there have been studies documenting the experience of grief (e.g. Arnold, Buschman Gemma, & Cushman, 2005), there appears a dearth of research on how individuals subjectively experience grief therapy (Breen & O’Connor, 2007).

The purpose of the current study was to assess participants’ experiences of therapy following the death of a loved one and whether CBT or EMDR resulted in common or unique experiences. The study aimed to explore the following issues: what are the similarities and differences between CBT and EMDR in terms of the participant’s experiences of the therapeutic relationship, perceived helpful and unhelpful processes in therapy, and perceived outcomes?

## 1. Method

### 1.1. Design

Participants who identified themselves as experiencing difficulties in coping with the death of a loved one were randomly allocated to a therapist and treatment condition (CBT or EMDR) and one of two therapists (Meysner et al., 2016). Following treatment they were interviewed by a clinician who had not provided any treatment to the participants. The resultant semi-structured interview was recorded and an inductive thematic analysis of the interviews was performed in line with Braun and Clarke’s (2006) guidelines. The study was approved by a University Human Research Ethics Committee. All participants were informed about the interview process via an information sheet and given an opportunity to ask questions. All agreed in writing to participate.

### 1.2. Participants

Nineteen participants (12 females and 7 males), aged between 22 and 75 years (*Mean = *45.6, *SD = *15.52), volunteered to participate in the study, and all but one completed treatment. The one participant who did not complete received EMDR and could not be contacted after the fifth session. No individual declined to be interviewed. The remaining 18 participants were interviewed at the university psychology clinic where the therapy occurred. Nine received CBT and 9 received EMDR.

Time since the death of a loved one ranged from 6 months to 24 years (*Mean *= 5.5 years, *SD* = 7.9). Participants’ relationships with the deceased varied and can be seen in Table 1; causes of death also varied and included miscarriage, medical condition, motor vehicle accident and suicide.

**Table 1.** Type of relationship the participant had with the deceased.

	Spouse	Participant’s parent	Participant’s child	Girlfriend/ boyfriend	Other family
Proportion *(n)*	42.1 *(8)*	31.6 *(6)*	10.5 *(2)*	5.3 *(1)*	10.5 *(2)*

Participants were recruited through referrals from community clinics and local general practitioners, as well as advertisements placed in local media and posters displayed around the university campus. Eligibility criteria included being over the age of 18 years and having lost a significant person through death at least six months prior.

### 1.3. Therapists and fidelity

The treatment involved one of two therapists and each administered both treatment types. Both therapists were postgraduate clinical psychologists and attended external training programmes in CBT and EMDR therapies. Each therapist was found to have satisfactory fidelity to each treatment condition (for details see Meysner et al., 2016).

### 1.4. Interventions

EMDR sessions followed an eight-phase protocol originally described by Shapiro in 1995. Therapists used the first session to explore the participant’s loss and grief experiences, provide psycho-education on EMDR and teach a ‘safe place’ imagery exercise. Therapists followed Luber’s protocol (2012) in sessions two to six and addressed distressing targets in the following order: past memories; intrusive images; nightmares; present triggers; and creating a future template. Typical targets included memories of the loss of the significant person, such as being notified of the death or the funeral. Therapists used session seven to summarize and reflect on the experiences of therapy, to problem solve any remaining concerns and to discuss future options for support.

The CBT protocol was based on the integrative cognitive behavioural treatment manual for grief developed by Rosner, Pfoh, and Kotoucova (2011). Similar to the EMDR condition, the first session involved psycho-education on grief and CBT, exploration of the participant’s unique grief, and the teaching of relaxation exercises. Sessions two to six then addressed the following elements in sequence: identifying triggers, unhelpful thoughts and emotional reactions; challenging unhelpful thoughts; an imagery farewell ritual; and addressing and confronting avoidance. Therapists used the seventh session to summarize and reflect on the course of therapy and provide information on further supports available.

### 1.5. Interviews

After completing therapy, participants were contacted via telephone by a third therapist within two days to arrange a time to attend a face-to-face interview. The mean time between finishing treatment and attending the interview was 16 days (range: 7–28). This semi-structured interview was conducted by a person who was not the participant’s therapist. It was designed as an empathic exploration of each participant’s experiences of therapy. Examples of the questions asked and the response prompts used are listed in Table 2.

**Table 2.** Questions and follow-up prompts used in the interview.

Broad question	Prompts if needed
Can you start by telling me about the therapy you received and your thoughts about it?	What part of the therapy did you like the most?
	What part of the therapy did you dislike the most?
	What was the most helpful/memorable part of your therapy?
	What was the least helpful or memorable part of your therapy?
Now we have discussed the therapy, I would like to discuss how you experienced the therapeutic relationship. What were your thoughts about your therapist *(insert therapist name)?*	How comfortable did you feel in working with her?
	Did you feel that your relationship with *(insert therapist name)* had any impact on the therapy you were given?
	How so?
Can you tell me what happened over the time you received therapy?	If any of the following areas are not mentioned in response then prompt for these and follow with ‘Tell me more about this’ only if given affirmative responses:
	Have you noticed any changes in your memory of your loved one?
	Have you noticed any changes in your thoughts or feelings about your loved one?
	Have you noticed any changes in what you do since the therapy?
	Have you noticed any changes positive or negative in any other area of your life?

Participants were interviewed, on average, for 21 minutes (range 11 to 54 minutes). Open and semi-structured format questions allowed therapists to adapt and elaborate flexibly, depending on the context. This dynamic process allowed interviews and data analysis to be conducted concurrently. Emerging themes from the data analysis were used to adjust the interview schedule. For example, it emerged that participants were able to identify components of the therapy that they disliked, but acknowledged that these were helpful (such as visualising the deceased’s body); therefore, prompts were adjusted to ask about what participants liked/disliked as well as what they found helpful.

### 1.6. Data analysis

All interviews were audio-recorded and transcribed. These transcripts were analysed in line with Braun and Clarke’s (2006) guidelines to thematic analysis. First, one of the authors (PC) read the interviews repeatedly to become familiar with their content. Segments of the text were then labelled and an initial list of themes were identified. These themes were discussed with the other authors (CL and LM) after six interviews. As each subsequent interview was analyzed, further additional themes were identified by PC. At the completion of all interviews, two of the researchers (PC and LM) met to review the themes and at that time identified higher order themes. They then further collated relevant text transcripts into these various categories. No new themes were identified after reviewing the first 14 transcripts. These themes were then reviewed to ensure coherency, distinctiveness and accurate reflection of the data by the research team (CL, HC, LM and PC). Once this process was complete a comparison of the resultant themes from each condition was conducted. To ensure the reliability of the analysis a reflective diary was kept (PC). The final coding scheme was then also checked with the interviewer (TM) who agreed with the end result.

## 2. Results

The main emerging themes from the data centred on outcomes experienced by the participants rather than the experiences of the processes in the treatment. Data analysis revealed the conditions shared many similar outcomes but there were also unique outcomes. The participants’ experiences are illustrated below with quotations, each with a participant identifier and indication of the treatment condition (e.g. CBT,1 or EMDR,7)

### 2.1. Similar themes found in both treatment conditions

**Figure 1.**
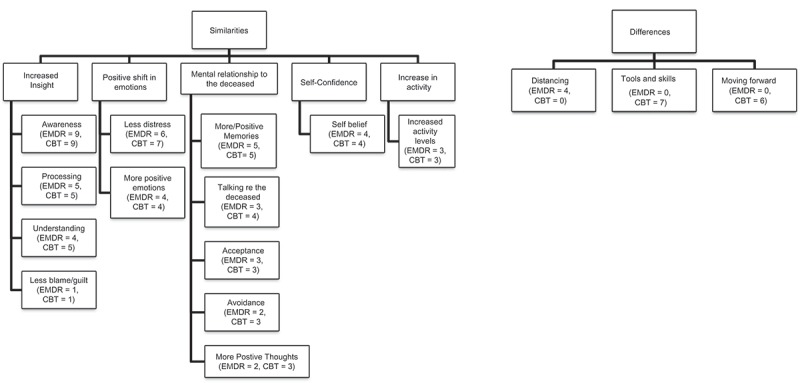
Themes organized by similarities or differences and then any overarching themes with participant’s condition and number of responses for each theme in brackets.

Analysis revealed 13 themes which were present in both conditions (see Figure 1.). These themes common to both conditions were categorized into five higher order key themes: insight; positive shift in emotions; mental relationship to the deceased; self-confidence; and activity levels. Figure 1 also shows the number of participants from each condition that endorsed each theme. This has been included to enhance transparency of the analysis and to allow the reader to observe the relative strength of each theme. Since these results refer to the similar outcomes demonstrated by both EMDR and CBT conditions, the discussion of the themes is not split between the two conditions.

#### 2.1.1. ‘Increased insight’

This theme concerns the participants’ development of an understanding of themselves, their grief and their reactions. On the most basic level, most participants reported that they became more aware of their thoughts and feelings: ‘It taught me things that I didn’t know before about my thinking’ (CBT, 6). For some, insight began with realizing that they had issues that needed addressing:

I didn’t really realize I had such a problem until it clicked and then I realized gee I did have a problem and it made me reassess my whole life … it was just like a light globe went on and I realized then exactly what was happening. (CBT, 3)

For others who already accepted their difficulties, it helped clarify from where their problems stemmed:

The main thing that I realized was that I had to do it … and that I had not been participating in the sort of things that I needed to, in order to deal with this grief situation … One was, talk to people about it. (EMDR, 4)

For several participants, the process of exploring their grief fostered an understanding of how they had come to deal with their loss, while some were able to make links between their grief and other life events. For example, when one participant was asked about the most helpful aspect of therapy, she replied: ‘I think me deciding that that was the cause of everything that happened in my life … And why I’ve made the decisions that I have made’ (EMDR, 15).

Participants reported that as they became more aware of their thoughts about their grief, they learnt to process and work through them. Examples of feedback included: ‘I guess it was the ability to identify the thought processes that were holding me back … then I guess the ability to sort of work through that’ (EMDR, 7) and ‘just coming to terms with seeing things in a different perspective, which then helps your thought processes to go in a more healthy direction’ (CBT, 16). Finally, being able to put things into perspective allowed two participants to feel a release from blame and guilt:

I was blaming myself so much for mum’s death. I had a very specific idea I think of what mum thought and what she would have said and I think by doing that exercise [imaginal conversation with the deceased] and actually having a different point of view I realized that maybe I was being too hard on myself and mum probably would actually see it that way. So I think it helped to lift a little bit of that burden of blame. (CBT, 1)

#### 2.1.2. ‘Positive shift in emotions’

This theme encompasses changes experienced by participants with regard to their emotions. Firstly, participants reported a decrease in distress, wherein negative emotions became less intense: ‘It helped a lot with the bitterness and the envy for the other people’ (CBT, 11). They also identified that these negative emotions became less frequent in their day-to-day lives:

There’s 7 days a week right? Previously it’s like, 5, like 6 days are horrible days and probably one day is a good day. But now, maybe … 4 days are good days, 3 days are bad days. (EMDR, 2)

As the distress decreased, it appeared to make way for more ‘positive’ emotions and outlooks (CBT, 11). Participants reported feeling ‘happier’ (CBT, 9), ‘calmer’ (EMDR, 8) and noticing an improvement in their ‘attitude’ (EMDR, 4).

#### 2.1.3. ‘Mental relationship to the deceased’

This theme encapsulates the participants’ improved ability to experience, tolerate, enjoy and share their thoughts and memories of the deceased. The nature of their thoughts surrounding the deceased appeared to shift from predominantly distressing thoughts, to appreciation and greater acceptance.

Many participants reported that they became more willing and able to sit with thoughts and painful memories of the deceased. By doing so, these events became integrated into their life stories and the distress associated with them decreased. As this occurred, participants reported increased recollections of memories of the deceased, and, in particular, positive memories:

Now I can remember her as she should be remembered, as a happy, fun loving mother of two that loved me to pieces and umm yea instead of the person who contracted a terrible disease and ended up weighing 20 kilo on the day she died … those thoughts or those memories don’t overtake anymore. I can look back now on the fun times we had and remember them. (CBT, 3)

Participants also expressed greater readiness to be around others as they spoke about the deceased. This allowed them to share their memories.

I think my family would see that I’m feeling a lot more positive about the memory of [my daughter], which helps, which helps them as well. Because they used to want to talk about it and you know, I’d just get angry. (EMDR, 8)

Interviewees from both CBT and EMDR conditions reported a greater sense of acceptance surrounding their loss. This included accepting the reality of the loss itself, as well as their own behaviours and reactions to the deceased person’s life and death.

I had a lot of trouble thinking about the hospital. I was with [my wife] when she died and she was as good as gold, and ah, then she just died. She was talking to me, and she said … ‘Nuh, I wanna go home, the pressure’s too great’. What she meant by that I’ll never know, and she died. And ah, she [therapist] made me accept that. (EMDR, 12)

In addition participants reported decreased avoidance of physical reminders of the deceased. ‘I’ve taken the photo album home and kind of, not hidden away as much as it was before, it’s kind of almost in plain sight’ (EMDR, 15).

Participants also described more positive thoughts about the deceased: ‘ … shifted again to a, to a more positive spin that I know that they are … . So, I think I’ve learnt that I can’t change what’s happened so why think in the negative about him’ (CBT, 16). At times this seemed to involve a broader understanding of the deceased and their actions: ‘More regret and sadness and a lot more pity and understanding of, just because he did a shit job doesn’t mean he wasn’t doing the best he could’ (EMDR, 18).

#### 2.1.4. ‘Self-confidence’

In both conditions, participants reported increased self-confidence and self-efficacy. This reflected another important shift in participants’ day-to-day functioning after treatment. Not only did they perceive themselves better able to cope with their grief, but the challenges of life in general. ‘It made me feel a lot stronger and confident in facing my feelings … it’s a very worthwhile technique and it did give me a lot of confidence’ (CBT, 22). ‘I feel more calm and self-assured. And, more capable I think. More in control as well … just more capable of … dealing with life and facing challenges’ (EMDR, 18).

In some participants, an increased feeling of self-worth was also described. One participant from the CBT condition described how she had overcome much of her negative self-talk, learnt to accept praise from others, and was more able to acknowledge and celebrate her own achievements as opposed to dismissing them as she had done previously. Another participant from the EMDR condition reported improvements in his feelings of self-worth, describing that the therapy helped most with ‘promoting tolerance and the feelings of worthiness’ (EMDR, 18).

#### 2.1.5. ‘Increased activity levels’

As they began to move through their grief, participants began to re-engage with hobbies and activities they previously enjoyed: ‘Yea I think I’m a little bit less sedentary, I do get off my backside and do things’ (EMDR, 4).

Right on the last session when we sort of worked out well how am I going to sort of start getting in to life again, and so we had a list of stuff that I had to do. So one of them was get back in to Karaoke and I’ve done that twice since with a new group of people. And I’m going skydiving on Saturday. And I did archery on Sunday. (CBT, 11)

### 2.2. Different themes found in EMDR and CBT treatment conditions

The analysis also revealed that there were key differences following treatment between the two conditions. As seen in Figure 1, participants in the EMDR condition reported feeling distanced from distressing memories. Participants in the CBT condition described the acquisition of tools for emotion regulation and moving forward.

#### 2.2.1. ‘Distancing of memories’

This theme refers to the decreased vividness with which distressing memories were recalled after therapy, and the accompanying decrease in distress caused by them. Participants described images as harder to visualize and being viewed from further away. Most significantly, the distress associated with these memories decreased allowing participants to notice these memories without becoming adversely affected by them: ‘Negative memories I tend to remember with less hatred and anger and they feel less directly emotive as though they weren’t happening to me, more empathetic in that I’ve seen it happening to someone else’ (EMDR, 18). This outcome was unique to the EMDR condition.

#### 2.2.2. ‘Tools and skills’

Participants in the CBT condition reported that they had acquired ‘tools’ and ‘skills’ that enabled them to regulate their emotions. As part of the CBT protocol, two sessions were dedicated to identifying unhelpful thoughts, linking these to distressing emotions, then challenging these thoughts and producing alternatives that reduced distress.

The new thought patterns that I’ve learnt to use, I think has been the biggest change. I can put aside those negative, depressive thoughts now and change it with another one and that’s the key to this whole therapy, is that it gives you, you learn an alternative thought and that’s what lets you go on to improving yourself and getting over that. (CBT, 6)

This is similar to ‘processing’, an outcome code shared by both conditions. The distinctiveness of this theme lies in the CBT participants’ description of doing this with the use of a ‘tool’. Whereas processing refers more generally to an overarching ability to notice thoughts and to churn through them, the CBT participants reported that they had a deliberate method for doing this; it was under their control and they were responsible for the benefits achieved. Furthermore, the CBT participants reported that this method for consciously regulating their emotions was helpful in other aspects of life, not just grief. Those in the EMDR condition did not report any such generalization.

#### 2.2.3. ‘Moving forward’

This theme reflects a perspective shown in some of the CBT participants to look at the next stage of their life, beyond grief. With it, came the possibility of enjoying that life. These participants recognized that they had made progress and were beginning to look beyond grief. ‘I’ve been given the tools to be able to work through that and move on to the next phase … now I’m looking forward to the next stage of life and whatever that brings’ (CBT, 3). ‘I’m done surviving and I’ve started living. And there’s so far to go with the goals and things like that, but I’ve got goals to look forward to, so yeah, daily life is quite different’ (CBT, 11).

## 3. Discussion

The results of the study demonstrate similar and disparate perceived outcomes for participants receiving CBT or EMDR for grief. For both treatment conditions participants reported that therapy enabled them to gain insight, experience a positive shift in emotions, develop and improve their on-going mental relationship to the deceased, enjoy improved self-confidence and become more active. These themes appeared with equal prevalence in each condition.

All participants discussed improvements in insight, facilitated by the therapy. In particular they described an improvement in self-awareness. Developing insight and awareness has been recognized as important in the treatment of grief with interventions developed that focus on participants developing insight (Piper, Ogrodniczuk, Joyce, McCallum, & Rosie, 2002). These reported experiences in both conditions is not surprising given that CBT has been linked with improved insight in the past (Holtforth et al., 2007) and that EMDR has been reported to improve self-awareness (Shapiro, 1995).

Another outcome expressed by participants in both conditions related to shifting emotions; they reported a decrease in distress and an increase in positive emotions. This is consistent with the quantitative data in the study, which demonstrated reductions on measures of distress (Meysner et al., 2016), and is also consistent with previous research on grief therapy, demonstrating improvements in depression and anxiety following treatment (e.g. Shear et al., 2005). The finding in this study of improvements in positive emotion is consistent with research on the process of healthy grieving, which indicates that with time, negative emotions become less intense and more manageable, as positive emotions begin to resurface (Worden, 2008). Furthermore, in a prospective longitudinal study of participants who had lost a loved one, the presence of positive emotions at various time points was associated with better outcomes in terms of social interactions and levels of distress (Tweed & Tweed, 2011).

Participants from both treatment conditions reported changes in how they experienced thoughts and memories of the deceased. They expressed greater understanding of their loved ones and the circumstances surrounding their deaths. Many reported reconnecting with positive memories of the loved one. The finding that positive memories began to resurface is consistent with previous studies using CBT (Maccallum & Bryant, 2011) and EMDR (Sprang, 2001) for grief.

The belief that one cannot function without the deceased is a proposed criterion for PCBD. Following treatment, participants in this study reported improved confidence in their ability to cope with grief and life’s challenges in general.

Whilst both conditions reported improvements in their emotions, CBT participants reported that they had developed specific skills for regulating their grief and other emotions such as anxiety. This outcome has been identified in other qualitative research and has been referred to as the ‘tool kit’ provided by CBT (Gega et al., 2013). In contrast, participants in the EMDR condition did not describe the acquisition of tools that could be used after therapy; previous research has described this as a limitation of EMDR (Maxwell, 2003). Interestingly, despite less of an active role in ‘doing’ tasks in EMDR, and not taking away tools to manage in future, EMDR participants still reported increased confidence in their ability to cope following therapy.

Another set of reported experiences that were unique to CBT participants was the theme of moving forward. They reported a sense of shift from grief to the next stage of life which was associated with hope and enjoyment. One possible explanation for this difference between conditions is the different focus placed on the future by the two therapies. Whilst EMDR addressed future obstacles, it did not build future goals. In a sense, EMDR taught participants that they could get through difficult times, whereas CBT promoted active work towards building good times.

Distancing from distressing memories was an outcome unique to EMDR. Participants reported that these memories became less vivid, harder to visualize and triggered less emotion. This is commonly seen when using EMDR to treat PTSD (e.g. Lee, Taylor, & Drummond, 2006) and according to the Adaptive Information Processing (AIP) model is evidence that memories are becoming integrated with broader memory networks (Shapiro & Laliotis, 2011). The AIP model suggests that instead of being stored in a narrative or semantic form, traumatic memories are stored in what has been described as a ‘here and now’ or episodic form. Therefore they continue to be recalled with high levels of distress and experienced as flashback-like sensations (Shapiro & Laliotis, 2011). The goal of EMDR is to access these memories and to stimulate the assumed innate information processing system to assist in linking these memories with existing more positive memories. This results in the negative memories becoming less episodic in nature and therefore may be recalled without significant distress (Shapiro, 1995). Key memories associated with grief share characteristics with traumatic memories, including vivid content and strong, distressing emotions, such as an argument with the deceased prior to their death or watching the coffin being lowered into the ground. These intrusive memories are targeted in EMDR and in the present study, participants reported that they became more distant. Of note, they also then reported positive shifts in emotions, improved self-confidence and became increasingly active even though these changes were not specifically targeted in the therapy.

Other researchers have also proposed that in traumatic grief, memories of a lost one are poorly integrated into the broader memory network (Smid et al., 2015). In proposing an eclectic therapy designed specifically for treating grief, they stated that the process of facilitating such an integration was a key to recovery.

### 3.1. Strengths and limitations

This was the first qualitative study to explore the experiences of individuals’ grief therapy. We chose not to limit our investigation to only participants who met a formal diagnostic criteria (PGD or PCBD) for grief keeping in line with the exploratory nature of the study. This might limit the generalizability of the findings to those that do meet such criteria. In a recent meta-analysis, it was found that the greatest benefits of grief therapy are achieved by those who meet a diagnostic criteria for a grief disorder (Wittouck et al., 2011). However, a previous meta-analysis compared studies where participants identified that they needed help with grief to those who were at risk of developing pathology and found that when a person sought help the effects of therapy were larger (Allumbaugh & Hoyt, 1999). Given the possibility that having a diagnosis may affect how the therapy is experienced, then it would be interesting to see if the current findings could be replicated in a study where only those with a grief disorder were included. Alternatively both groups of participants could be included and assess whether a diagnosis mediated the findings reported by participants.

Another potential limitation is that participants were not contacted to provide feedback on the findings.

### 3.2. Conclusions and recommendations

Following each treatment, participants reported improvements in wellbeing such as greater insight, more positive emotions, a change in how they view the deceased, improvements in self-confidence and an increase in activities. There were also unique benefits reported for each condition. For those receiving EMDR it was a greater distancing from distressing memories. This finding of an improvement in distancing and an increase in wellbeing is consistent with the AIP model which has been hypothesized to underlie EMDR. On the other hand, a unique aspect of the CBT treatment was the acquisition of emotion regulation skills that participants reported they could use in many areas of their lives. This is consistent with the idea that CBT provides a tool kit for people experiencing distress associated with grief.
